# Violent Infant Surrogate Shaking: Continuous High-Magnitude Centripetal Force and Abrupt Shift in Tangential Acceleration May Explain High Risk of Subdural Hemorrhage

**DOI:** 10.1089/neur.2021.0013

**Published:** 2021-05-26

**Authors:** Arne Stray-Pedersen, Frode Strisland, Torleiv Ole Rognum, Luuk Antoon Hubertus Schiks, Arjo Jozef Loeve

**Affiliations:** ^1^ Department of Forensic Sciences, Division of Laboratory Medicine, Oslo University Hospital, Nydalen, Oslo, Norway.; ^2^ Department of Forensic Medicine, Institute of Clinical Medicine, University of Oslo, Blindern, Oslo, Norway.; ^3^ SINTEF Digital, Department of Health Research, Oslo, Norway.; ^4^ Department of Biomechanical Engineering, Faculty of Mechanical, Maritime and Materials Engineering, Delft University of Technology, Delft, The Netherlands.; ^5^ Co van Ledden Hulsebosch Center of Forensic Science and Medicine, Amsterdam, The Netherlands.

**Keywords:** abusive head trauma, biomechanical model, infant, injury, shaken baby

## Abstract

Violent shaking is believed to be a common mechanism of injury in pediatric abusive head trauma. Typical intracranial injuries include subdural and retinal hemorrhages. Using a laboratory surrogate model we conducted experiments evaluating the head motion patterns that may occur in violent shaking. An anthropomorphic test device (ATD; Q0 dummy) matching an infant of 3.5 kg was assembled. The head interior was equipped with accelerometers enabling assessment of three-axial accelerations. Fifteen volunteers were asked to shake the surrogate vigorously holding a firm grip around the torso. We observed the volunteers performing manual shaking of the surrogate at a median duration of 15.5 sec (range 5–54 sec). Typical acceleration/deceleration patterns were produced after 2–3 shakes with a steady-state shaking motion at a pace of 4–6 cycles (back and forth) per second. Mean peak sagittal tangential accelerations at the vertex were 45.7*g* (range 14.2–105.1*g*). The acceleration component in the orthogonal direction, the radial acceleration, fluctuated around a negative mean of more than 4*g* showing that the surrogate head was continuously subjected to centripetal forces caused by rotations.

This surrogate experiment showed that violent shaking may induce high peak tangential accelerations and concomitantly a continuous high-magnitude centripetal force. We hypothesize that the latter component may cause increased pressure in the subdural compartment in the cranial roof and may cause constant compression of the brain and possibly increased stretching or shearing of the bridging veins. This may contribute to the mechanism accountable for subdural hematoma in abusive head trauma.

## Introduction

Abusive head trauma (AHT) is a major cause of morbidity and mortality in infants and young children.^1–4^ The injury mechanisms are controversial and include various forms of violence such as blunt force impact, shaking, and compression.^1^ Victims of AHT may present with subdural hemorrhages, retinal hemorrhages, and various degrees of encephalopathy, often with absent or inconsistent history, and commonly accompanied by other injuries indicative of abuse, such as fractures and bruises.^3,4^ Signs of direct impact to the head are lacking in a large proportion of the victims. This clinical evidence along with the sometimes questionable admissions by perpetrators, form the basis of the hypothesis that violent shaking alone, without impact to the head, may cause severe intracranial injuries.^5^ Such cases could be referred to as inflicted head injury by shaking trauma (IHI-ST).^6,7^

Biomechanical experiments with manual shaking of surrogates/dummies equipped with sensors have been performed in previous studies, but the accelerations measured have been below the commonly applied injury thresholds for impact trauma in adults.^8–11^ However, impact trauma differs largely from violent shaking events, especially concerning the duration of the acceleration peaks and the repeated loading due to the cyclic motion. Further, the loads generated by an impact depend on various factors, such as the elastic properties of the surrogate's surface and skull and the impact direction. Therefore, it is highly questionable whether using impact thresholds on non-impact repeated shaking is valid.

Previous biomechanical surrogate experiments applied simple neck joints such as hinges or short rubber necks. The accelerometers used were usually uniaxial in the anterior-posterior plane^8,10^ or were triaxial but without reporting of the accelerations in all directions.^11^ Estimates of rotational accelerations were provided based on calculations that ignore the fact that not only does the head rotate with respect to the body, but the body also moves and rotates through space. Yet, such measurements are, from a kinematics point of view, essential to fully grasp the mechanisms potentially underlying IHI-ST. To our knowledge, no biomechanical experiments that measured the head motion pattern and linear accelerations involved in violent shaking in three dimensions (three dimensional [3D]) have been reported in the literature. The aim of the present study was to quantify the 3D linear accelerations in repeated shaking to explore possible injury mechanisms.

## Methods

### Surrogate

**FIG. 1.**
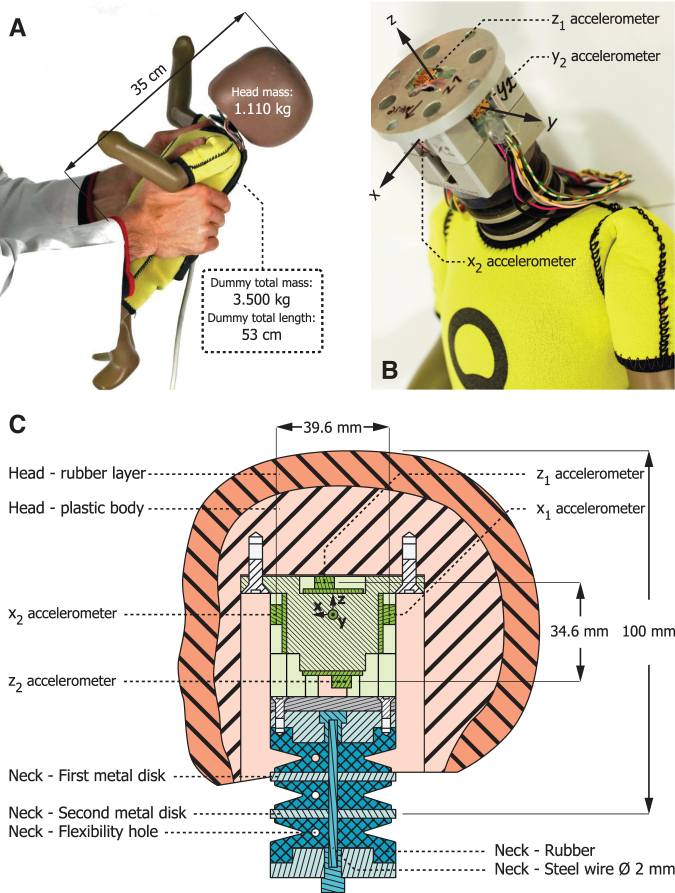
The instrumented Q0 dummy used for the shaking experiments. **(A)** Dummy in the hands of a participant. **(B)** Sensor module inside the head containing two accelerometers on each (x, y, z) axis. Positive (x, y, z) axes are indicated with arrows. **(C)** Schematic drawing of neck, dummy head.

An anthropometric test device (ATD), a human surrogate proportionate to a 1-month-old infant (Fig. 1), Q0 dummy was purchased from First Technology Security Systems in Delft, The Netherlands. It was originally developed for automotive passenger-safety crash tests and weighs 3.500 kg in total, with the head weighing 1.110 kg. Full body length is 53 cm, with a crown-rump length of 35 cm. The surrogate is designed to be biofidelic in its response to high accelerations based on extrapolations from adult biomechanical properties. The flexible neck consists of three rubber sections with two intermediate metal discs, allowing bending of the neck in all anterior-posterior and lateral directions, being less resistant to movement in the anterior-posterior plane than in the coronal plane. The distance from the vertex to the second metal disc is 10.0 cm.

The axis of the neck contains a 2-mm diameter steel wire for limiting the neck movements within biofidelic borders. The surrogate was calibrated and tested by the manufacturer before the experiment.

### Instrumentation

The surrogate head contains a centrally placed cubic metal bracket providing the head weight. The modified bracket holds six uniaxial accelerometers (Type 1212J-400, manufacturer-calibrated, measurement range: 400*g*; Silicon Design Inc.), one on each side (Fig. 1). These sensors were mounted on custom-made printed circuit boards with power regulators for limiting the effect of power supply variations on the measurements (Fig. 1).

The sensors measure acceleration in *g* ( = 9.81 m/sec^2^) in the direction orthogonal to their flat side and were arranged to measure in a right-handed coordinate system: The two accelerometers measuring anteroior-posterior movement (X1 and X2) were mounted on the frontal and occipital side of the bracket, providing positive measurements in the forward direction (occiput to nose); accelerometers Y1 and Y2 were mounted on the lateral sides, providing measurements in a positive direction from right to left; and accelerometers Z1 and Z2 were placed on the top and bottom of the bracket, providing measurements in a positive direction upwards. Using paired sensors placed spatially apart on the same axis improves the measurement robustness, allowing easy detection of any eventual sensor failure, and allowing determining rotations. A seventh uniaxial accelerometer was mounted on the vertex of the head (not shown in Fig. 1), providing measurements of anterior-posterior accelerations in a positive direction forwards. The cables from the accelerometers were passed individually from the head to the body of the surrogate, thereby leaving the head mechanics and neck stiffness essentially unaffected.

### Data acquisition and calibration

The accelerometers signal wires exiting the surrogate torso were bundled into a ∼10-m long, 7-mm thick, flexible plastic-coated cable running to the external data logging system: a dedicated National Instruments (NI) LabView program and a NI USB 6251 Data Logging Device.

Accelerations were sampled at ∼1 kHz, which was well below the accelerometers' maximum measurement frequency, and were stored in raw, unfiltered format during the experiments. The measurement sensitivity was ∼0.1*g*, slight absolute acceleration drift. Before measurements, the setup was calibrated by successively aligning the three measurement axes with gravity and adjusting their offset to display 1*g*. Intermediate brief drop tests displayed only marginal differences between the readings of any pair of sensors.

### Laboratory experiment

Nine male and six female volunteers, 35 to 65 years of age, were asked to hold the surrogate with their hands in a firm grip around the torso and shake as violently and as long as they could. All volunteers shook face-to-face with the surrogate. Each volunteer performed two consecutive shaking tests. Before each test, the setup calibration was confirmed by putting the surrogate horizontally on a flat table. The experiments were video-taped using a camera at 25 frames/sec. For each volunteer, the test run with the highest two consecutive peak vertex accelerations was selected for further analysis to find a reliable worst-case scenario. All volunteers agreed with publishing the gathered data.

## Results

**FIG. 2.**
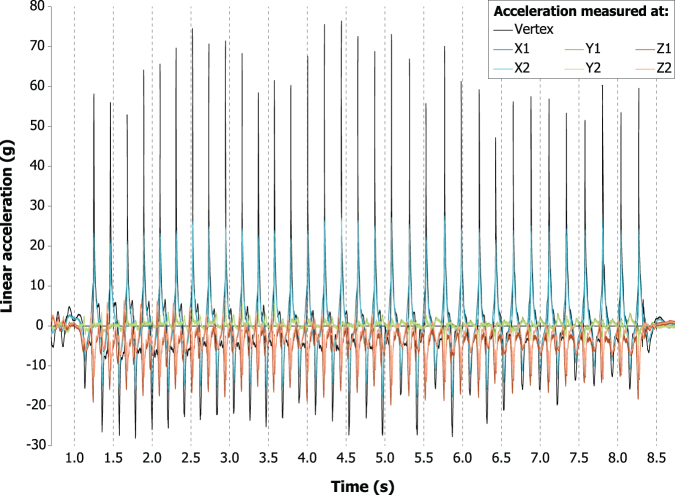
Accelerations measured by the vertex, x, y, and z sensors during the entire shaking session of Volunteer 2.

The mean duration of vigorous surrogate shaking performed by the volunteers was 15.5 sec (range 5–53 sec), with a mean back-and-forth shaking frequency of 4.6 Hz (range 3.5–6.0 Hz; Table 1). Acceleration patterns typically steadied after 2–3 shakes and showed little variation throughout each test, as illustrated with the data of Volunteer 2 in Figure 2. The mean peak tangential acceleration at the vertex was 45.7*g* (range 14.2–105.1*g*). The mean peak accelerations in the skull center were 18.6*g* (range 6.5–36.2*g*) in the X-direction, 4.9*g* (range 2.5–11.7*g*) in the Y-direction, and −17.5*g* (range −5.2 to −44.0*g*) in the Z-direction.

**Table 1.** Maximum Linear Accelerations, Shaking Frequencies, and Shaking Durations Measured with the Instrumented Surrogate Doll during Violent Shaking by Volunteers

	Shaking		Maximum linear acceleration (*g*) per direction			
Volunteer	Duration (sec)	Frequency (cycles/sec)	Vertex	X	Y	Z
1	8	4.5	76.5	27.6	6.1	−19.8
2	19	5.1	105.1	36.2	−10.2	−22.6
3	14	4.6	19.9	8.5	2.9	−6.7
4	10	4.6	40.4	14.6	−4.6	11.2
5	13	6.0	66.0	23.7	−11.7	−23.4
6	12	4.5	49.1	18.5	6.7	−15.9
7	21	4.3	32.6	14.5	2.6	−13.1
8	20	5.3	−42.7	−21.9	3.4	−22.6
9	35	4.5	41.2	13.8	−4.1	12.7
10	12	4.6	49.1	19.0	2.9	−16.8
11	16	4.5	56.4	31.9	4.6	−44.0
12	9	4.3	14.2	6.5	−2.5	−5.2
13	28	4.2	−32.1	−11.5	4.4	−13.0
14	18	4.0	37.3	13.0	−3.7	−17.4
15	19	3.5	22.4	17.7	3.6	−17.5

In the tests with Volunteers 3 to 15 the neck cable (2-mm steel wire, see Fig. 1) of the dummy was broken.

**FIG. 3.**
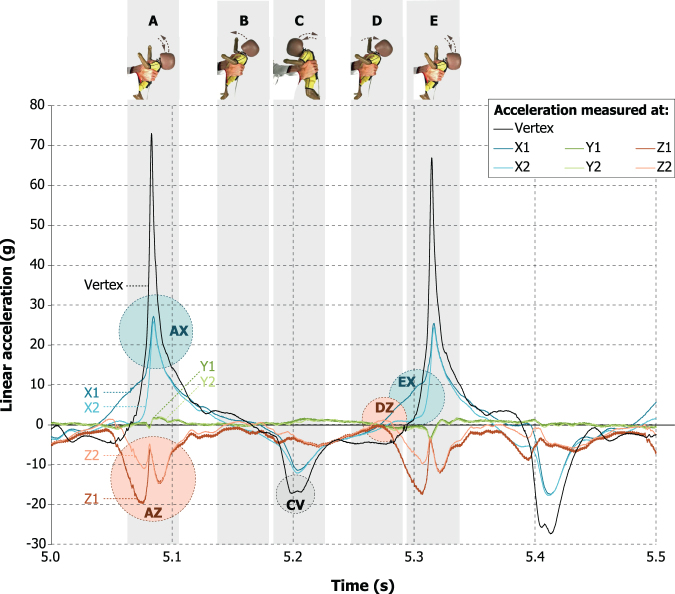
Detail view of seconds 5.0 to 5.5 from Figure 2, the accelerations measured for Volunteer 2. Shaded bars A to E and the photos on top of these bars illustrate which accelerations were measured during which phase in the shaking cycle. The dashed arrows in A to E indicate the motion directions of the dummy head. Shaded circles AX, AZ, CV, DZ, and EX are regions of interest that are further discussed in the text.

The acceleration plots showed large asymmetry: The peak positive tangential acceleration (posterior-anterior X-direction) was up to 3 times larger than in the negative (backwards) direction, as illustrated in Figure 3 (AX and CV) for the two cycles between 5 and 5.5 sec in Figure 2. When comparing the video recordings, this phenomenon seemed to be related to the volunteer leaning forward while shaking, the peak acceleration force being generated when the volunteer had their arms stretched and the surrogate was pulled upwards again. In 11 of the 15 tests, the acceleration in the Z-direction (neck to vertex) remained negative throughout the majority of the shaking test, fluctuating around a mean of −4.8*g* and showing that the surrogate head was continuously subjected to centripetal forces caused by rotations.

It should be noted that during analysis it was discovered that after Volunteer 2 the measured values changed considerably. Further investigation revealed that the steel cable in the dummy neck had broken, which was assumed to have happened after Volunteer 2. Consequently, the first two volunteers showed considerably higher accelerations than the later volunteers. However, because this provided the opportunity to show the importance of proper neck and end-stop modeling (the relevance of which is explained in the Discussion section), it was decided to still use all data for further analysis.

## Discussion

The current study provided 3D linear accelerations of the head of a biofidelic infant-sized dummy being violently shaken. Depending on how volunteers shook the dummy in the current tests, the surrogate head experienced either a predominantly negative or even a constantly negative acceleration in the Z-direction. This is most likely because the shaking followed a largely rotational pattern. The continuous high-magnitude centripetal force observed during this shaking experiment may give significant clues to understand the injury mechanisms in IHI-ST.

Subdural hematoma in IHI-ST has traditionally been contributed to tearing of bridging veins caused by traction and shearing forces.^3^ Rotational motions have been shown to cause a diffuse pattern of strains in the brain^12^ and modeling studies have suggested that pressure buildup may contribute to both brain injury and retinal hemorrhages.^7,13^ In a real infant victim the consistently negative Z-acceleration during shaking is expected to drive the intracranial contents against the cranial periphery, causing an increased intracranial pressure to build up over the entire duration of shaking. The increased pressure may cause increased traction on the bridging veins and increased deformation of the brain, but the detailed mechanism depends on several factors. When the density of a brain is lower than that of its surrounding intracranial fluids, it is expected that the fluids will accumulate between the brain and the skull. As the partially submerged buoyant brain is being pushed in the direction away from the force, the cerebrospinal fluid layer thickness increases, which cause further stretching of the bridging veins. Yet, when the brain is denser than the fluids, the cerebrospinal fluid layer more likely decreases, reducing bridging vein stretching, but potentially increasing shear forces on the brain and bridging veins instead. So for modeling or analyzing IHI-ST, it is essential that anatomy and tissue properties are known and validated.

The accelerations observed in the Y-direction were always very low compared with the X- and Z-accelerations. Although it cannot be excluded that even small out-of-plane accelerations perhaps cause very specific and potentially harmful deformations of the contents of the head, this strongly suggests that simplifying IHI-ST as sagittal-plane-only motion, as is often done in IHI-ST modeling studies,^6,7^ is likely to be a good approximation, while greatly simplifying measurements and calculations. The X-accelerations showed typical cyclic patterns with motion directions being inversed at each cycle and peak accelerations being consistently high at these instances (Fig. 3, AX and EX). The motion direction changes most likely correspond with not only linear changes of direction but also rotational changes of direction, as the peak X-accelerations occurred close to a short diminishing of the Z-accelerations (Fig. 3, AZ and AX), suggesting a temporary diminishing of rotational velocity.

In the current study, no rotational accelerations or rotational velocities are reported. That is because straightforward kinematics show that based on the sensors in the dummy alone the instantaneous axes of rotation or acceleration cannot be adequately determined and thus neither can the rotational velocity or acceleration. Duhaime and colleagues used a dummy with an accelerometer on the vertex of its head measuring what would be the vertex acceleration in our experiments.^8^ From that, the rotational velocity and acceleration were calculated assuming the dummy head's center of rotation was in the neck. The resulting rotational velocity and acceleration were compared against impact trauma thresholds. They did not report actual peak accelerations, but *mean peak accelerations* over a period of 92–130 milliseconds around the actual peaks, being between 5.70*g* and 13.85*g* for flexible rubber, stiff rubber, and no-resistance hinge-joint dummy necks. This approach, also employed in other studies,^9,10^ ignores several potentially crucial issues:

1. The center of rotation may at some moments during shaking be the assumed one, but for the remainder of the time it may be much further away from or much closer to the center of the head. This is because not only does the head rotate around the neck during shaking, but the torso moves, rotates, and accelerates too. Hence, the actual instantaneous center of rotation for accelerations with respect to the earth constantly moves. This implies that the most (un-)favorable instantaneous center of rotation does not necessarily occur during the peak accelerations and values calculated by Duhaime and colleagues^8^ should not be interpreted as maximum or minimum values.
2. With the non-hinge necks, rotational speed and acceleration of the torso will be transferred to the head. However, this cannot be measured or calculated with a single sensor or a fixed rotation axis.
3. A single sensor setup cannot distinguish between linear and rotational accelerations.

To illustrate the importance of knowing the actual center of rotation, first the current peak vertex accelerations were used to calculate the peak angular velocity and acceleration in the same manner as by Duhaime and colleagues^8^ (Supplementary Table S1). Next, the peak angular velocity and acceleration were calculated for centers of rotation lower in the neck (0.1 m), at the volunteer's elbow (0.4 m), and much closer, in the center of the head—which could happen when the head continues rotating backwards while the torso is already being jerked forwards again (Supplementary Table S1). This showed that the volunteers in the current study shook as fiercely as was done in the study by Duhaime and colleagues.^8^ Yet more importantly, this illustrated that proper determination of angular components requires the actual center of rotation to be known at all instances of the shaking cycle. Hence, existing published data about the loads acting on infants during fierce shaking should be carefully interpreted.

Material tests on human infant bridging veins have shown that rupture occurs at much lower stresses after 30 sec of cyclic loading than after a single pull, and also that bridging veins break at lesser elongation for higher deformation rates.^14^ Combined with the consistently negative Z-acceleration shown in the current study, this may become a harmful cocktail during shaking: increased stress in the bridging veins due to increase blood pressure, combined with rapid loading, which further increases the stresses due to the viscoelastic response, and repetitive cyclic loading that leads to rupture at early stress levels. With the instantaneous center of rotation close to the center of the head, the measured accelerations lead to angular velocities and angular accelerations (see Supplementary Table S1) that are above the thresholds for 50% chance of extra-axial hemorrhage established by Pasquesi and associates in piglets.^15^ Human infant bridging veins seem to endure similar or slightly higher stretch and stress before rupturing, depending on the type of loading.^15^ This suggests that the measured accelerations could be in a harmful range if the transfer from skull kinematics to bridging vein loading is the same in infants as in piglets. However, the latter has yet to be investigated.

A potential error source in the present study is the biofidelity of the surrogate's neck. Several studies have shown that more compliant necks result in higher peak accelerations than stiffer necks.^8,10,16^ The surrogate neck in the present study is a scaled adult version that was designed to mimic the motion of a baby neck in high-energy trauma, such as car collisions. It is stiffer than in real infants, who possess little neck muscle tone and may not in such cases adequately support the weight of their heads. A highly flexible neck may allow more extreme head motion with respect to the torso at the end-points of the shaking motion. On the other hand, a stiffer neck transfers more of the rotational accelerations exerted on the torso to the head.

The measurements with the broken neck cable showed reduced peak accelerations but a rougher course of the accelerations in general (Supplementary Fig. S1). Without the constraining neck cable, head motions were more irregular. This may have also been due to the head bouncing when impacting the soft polyurethane and rubber layers of the doll and its suit. If true chest-chin or head-back impact is a hard contact, the neck cable may provide more realistic results, whereas if these contacts are relatively elastic or dissipative by nature, the situation without the neck cable would be more fitting. As neck stiffness has a considerable effect on head kinematics during shaking,^8,10^ all data were kept from the analyses, both due to uncertainty as to whether the intact neck cable was more realistic in terms of neck stiffness and impact, as well as to keep records of the changes that were made during the study.

Because a biofidelic neck, such as in the current tests, does not have a single axis of rotation, the location of the instantaneous axis with respect to the torso varies continuously during shaking and is not determined by geometric constraints alone. Further, during shaking the rotation with respect to the torso is at most instances not equal to the center of rotation with respect to earth, which determines the actual accelerations experienced by the dummy. Therefore, and because no orientation or position measurements were done, it was impossible to reliably determine the instantaneous centers of rotation and acceleration for the dummy head during shaking. Yet, the extent to which a combination of angular velocity and angular acceleration is harmful depends on where the center of rotation is located.

As an example, consider the skull and brain as a cup and water, respectively. Then, consider the difference between the accelerations experienced by a cup of water at the center of a rotating disk and one on the outer edge of the same disk. Both cups and the water within will experience the same rotational acceleration and velocity, but the water in the outer cup will behave very differently from that in the center cup. The water in the outer cup will concentrate against the sides of the cup directed away from the disk center and against the rotation direction of the disk. As a consequence, the potential harmfulness of the measured accelerations could not be fully estimated in the current study. It should be noted that these limitations should also be kept in mind when assessing the value of the evidence provided by earlier shaking studies.

## Conclusion

Approximately 47 years ago Guthkelch^17^ and Caffey^18^ hypothesized that violent abusive shaking may result in severe head injury in infants and small children. Although much has been learned since then, the current results suggest that more research is needed to properly determine the detailed kinematics of infants during shaking and their effects on the infant's anatomy. Commonly used shaking injury thresholds have recently been called into question for various reasons.^19^ Many such thresholds have not been properly validated for applicability to infants, and few take into account the very different loading patterns found in shaking versus impacts. Although impacts may cause large impulse transfers by short duration, high-intensity peaks, shaking may transfer just as large or even larger impulses with lower maximum forces, but over longer and repeated cycles. The repetitive loading and constant negative Z-acceleration shown to occur during fierce shaking in the current study may yield clues to understanding the injuries observed in IHI-ST.

## Supplementary Material

Supplemental data

Supplemental data

## Abbreviations Used

3D: three dimensional
AHR: abusive head trauma
ATD: anthropomorphic test device
IHI-ST: inflicted head injury by shaking trauma
NI: National Instruments
