# Supplementary material for: Violent Infant Surrogate Shaking: Continuous High-Magnitude Centripetal Force and Abrupt Shift in Tangential Acceleration May Explain High Risk of Subdural Hemorrhage
Source: Neurotrauma Rep. 2021 May 26;2(1):224–31. doi: 10.1089/neur.2021.0013 (PMC8240836; doi:10.1089/neur.2021.0013)
Supplement: Supplemental data [file Supp_TableS1.docx]

**Supplement**

**Table S1:** Maximum angular velocities (ω) and angular accelerations (α) calculated using the data from Table 1 and using the very simplified formulae: $\omega=\sqrt{a_{Z}\cdot r}$ and $\alpha=a_{V}\cdot r$, with $a_{Z}$, $a_{V}$ and $r$ being the peak linear Z-acceleration, peak linear Vertex-acceleration and rotation radius. Please note that, as is discussed in the main text, this approach ignores several crucial aspects and hence **should not be used in practice**. The values in this table only serve to compare the current data to the Duhaime et al. results (8) and to show how the angular variables calculated from the measured linear accelerations depend on the location of the rotation axis.

| Rotation axis location: | High in dummy head | | Low in dummy head | | Second disk of dummy neck | | Volunteer’s elbow | |
| --- | --- | --- | --- | --- | --- | --- | --- | --- |
| Rotation radius (m): | 0.02 | | 0.06 | | 0.10 | | 0.40 | |
| Volunteer | Ang. Vel.  (rad/s) | Ang. Acc. (rad/s^2^) | Ang. Vel.  (rad/s) | Ang. Acc. (rad/s^2^) | Ang. Vel.  (rad/s) | Ang. Acc. (rad/s^2^) | Ang. Vel.  (rad/s) | Ang. Acc. (rad/s^2^) |
| 1 | 98.5 | 37523.3 | 56.9 | 12507.8 | 44.1 | 7504.7 | 22.0 | 1876.2 |
| 2 | 105.3 | 51551.6 | 60.8 | 17183.9 | 47.1 | 10310.3 | 23.5 | 2577.6 |
| 3 | 57.3 | 9761.0 | 33.1 | 3253.7 | 25.6 | 1952.2 | 12.8 | 488.0 |
| 4 | 74.1 | 19816.2 | 42.8 | 6605.4 | 33.1 | 3963.2 | 16.6 | 990.8 |
| 5 | 107.1 | 32373.0 | 61.9 | 10791.0 | 47.9 | 6474.6 | 24.0 | 1618.7 |
| 6 | 88.3 | 24083.6 | 51.0 | 8027.9 | 39.5 | 4816.7 | 19.7 | 1204.2 |
| 7 | 80.2 | 15990.3 | 46.3 | 5330.1 | 35.8 | 3198.1 | 17.9 | 799.5 |
| 8 | 105.3 | 20944.4 | 60.8 | 6981.5 | 47.1 | 4188.9 | 23.5 | 1047.2 |
| 9 | 78.9 | 20208.6 | 45.6 | 6736.2 | 35.3 | 4041.7 | 17.6 | 1010.4 |
| 10 | 90.8 | 24083.6 | 52.4 | 8027.9 | 40.6 | 4816.7 | 20.3 | 1204.2 |
| 11 | 146.9 | 27664.2 | 84.8 | 9221.4 | 65.7 | 5532.8 | 32.8 | 1383.2 |
| 12 | 50.5 | 6965.1 | 29.2 | 2321.7 | 22.6 | 1393.0 | 11.3 | 348.3 |
| 13 | 79.9 | 15745.1 | 46.1 | 5248.4 | 35.7 | 3149.0 | 17.9 | 787.3 |
| 14 | 92.4 | 18295.7 | 53.3 | 6098.6 | 41.3 | 3659.1 | 20.7 | 914.8 |
| 15 | 92.6 | 10987.2 | 53.5 | 3662.4 | 41.4 | 2197.4 | 20.7 | 549.4 |
