# Supplementary material for: Violent Infant Surrogate Shaking: Continuous High-Magnitude Centripetal Force and Abrupt Shift in Tangential Acceleration May Explain High Risk of Subdural Hemorrhage
Source: Neurotrauma Rep. 2021 May 26;2(1):224–31. doi: 10.1089/neur.2021.0013 (PMC8240836; doi:10.1089/neur.2021.0013)
Supplement: Supplemental data [file Supp_FigS1.docx]

**Figure S1:** Typical example of accelerations measured after the neck cable had broken. Accelerations measured for volunteer 11 between seconds 2.0 and 2.5. Note the irregular motions in comparison with Figure 3 before the neck cable broke.
